# Long non-coding RNA FENDRR regulates IFNγ-induced M1 phenotype in macrophages

**DOI:** 10.1038/s41598-020-70633-7

**Published:** 2020-08-13

**Authors:** Maria Cristina Munteanu, Chaoqun Huang, Yurong Liang, Roshini Sathiaseelan, Xiangming Zeng, Lin Liu

**Affiliations:** 1 Oklahoma Center for Respiratory and Infectious Diseases, Oklahoma State University, Stillwater, OK USA; 2 Lundberg-Kienlen Lung Biology and Toxicology Laboratory, Department of Physiological Sciences, Center for Veterinary Health Sciences, Oklahoma State University, 264 McElroy Hall, Stillwater, OK 74078 USA

**Keywords:** Cell signalling, Long non-coding RNAs

## Abstract

Macrophages play an essential role in host defense and display remarkable plasticity in switching between classically (pro-inflammatory—M1) and alternatively activated (anti-inflammatory—M2) phenotypes. The molecular mechanisms of macrophage polarization are not fully understood. Long non-coding RNAs (lncRNAs) with a length of > 200 nucleotides have been shown to play diverse roles in biological processes. Aberrant expression of lncRNAs is associated with a variety of pathophysiological conditions such as cancer, diabetes, cardiovascular, pulmonary diseases, and tissue fibrosis. In this study, we investigated the role of lncRNA FENDRR in human and mouse macrophage polarization. Human THP-1 monocytes were activated with phorbol-12-myristate-13-acetate (PMA) and differentiated into M1 macrophages with IFNγ or M2 macrophages with IL4. Real-time PCR analysis revealed that FENDRR was expressed 80-fold higher in M1 macrophages than that in M2 macrophages. Overexpression of FENDRR in PMA-activated THP-1 cells increased the IFNγ-induced expression of M1 markers, including IL1β and TNFα at both mRNA and protein levels. Knockdown of FENDRR had an opposite effect. Similarly, FENDRR overexpression in primary mouse bone marrow-derived macrophages increased mRNA expression of M1 markers. FENDRR overexpression increased, while FENDRR knock-down decreased, the IFNγ-induced phosphorylation of STAT1 in PMA-activated THP-1 cells. Our studies suggest that FENDRR enhances IFNγ-induced M1 macrophage polarization via the STAT1 pathway.

## Introduction

Macrophages play a central role in inflammation and host defense and are an essential component of innate immunity^1^. Upon activation, macrophages acquire diverse phenotypes and functions in response to microenvironmental signals. Depending on the stimulus and the microenvironment, macrophages can phenotypically differentiate into either “classically activated” M1 macrophages induced by IFNγ, LPS, and GM-CSF or “alternatively activated” M2 macrophages, driven by IL10 and IL4. M1 macrophages are characterized by a pro-inflammatory phenotype, showing increased expression of IL1β, TNFα and IL6^2^. Macrophage polarization has been described as an important component of many diseases, including fibrosis^3^, cancer^4^, infection^5^, insulin resistance^6^, atherosclerosis^7^, and autoimmune disease^8^. However, the molecular regulatory mechanisms controlling the expression of specific genes involved in macrophage polarization are not fully clear. The understanding of molecular mechanisms underlying macrophage plasticity and polarization will provide a basis for macrophage-centered diagnostic and therapeutic strategies.

Recently, non-coding RNAs (ncRNAs) have been described as key regulatory molecules, with diverse roles in fundamental biological processes^9,10^. Long non-coding RNAs (lncRNAs) play essential roles in many cellular and developmental processes, including cell proliferation, apoptosis, and differentiation as well as organ morphogenesis^11,12^. Furthermore, lncRNAs are important regulators of the immune response in monocytes and macrophages^13^. LncRNAs are usually divided into five categories: sense, antisense, bidirectional, intronic and intergenic.

A few studies has reported the involvement of lncRNAs in macrophage polarization. Using microarray analysis, Huang et al. revealed the expression profile of lncRNAs in monocyte-derived macrophages with polarized phenotypes^14^. Deregulated lncRNAs in polarized macrophages are mainly located in intergenic regions (50%), followed by antisense to protein-coding genes (35%). Further studies have shown that lncRNA TCONS_00019715 is expressed at a higher level in IFNγ and LPS-polarized M1 macrophages than in IL4-polarized M2 macrophages. Knockdown of TCONS_00019715 reduced the expression of M1 markers and increased the expression of M2 markers, suggesting that TCONS_00019715 promotes macrophage polarization to the M1 phenotype^14^. Sun et al. has identified lncRNA GAS5 as an epigenetic regulator of microglial (major innate immune cells in the central nervous system) polarization by inhibiting the transcription of TRF4 via recruiting the polycomb repressive complex 2 (PRC2)^15^. Ito et al. has also described GAS5 as a key factor involved in M2b (CCL1+ LIGHT+, IL10+) macrophage polarization, mediated by the activation of the nonsense-mediated RNA decay (NMD) pathway^16^.

Fetal-lethal non-coding developmental regulatory RNA (FENDRR) is an intergenic lncRNA. Mouse Fendrr is a 2,380 bp transcript consisting of six exons. It is transcribed from a bidirectional promoter shared with the protein coding gene Foxf1a, located 1,354 bp from its transcriptional start site. Loss of Fendrr is lethal in mice^17,18^. Fendrr is highly expressed in the adult lung and lowly expressed in the colon, liver, spleen and brain^17^. Fendrr is essential for proper development of tissues derived from the lateral mesoderm, specifically the heart and the body wall. Fendrr acts by modifying the chromatin signatures of genes involved in the formation and differentiation of the lateral mesoderm lineage through binding the PRC2 and Trithorax group/MLL (TrxG/MLL) complexes^18^. PRC2 catalyzes the methylation of histone H3 at lysine 27 (H3K27me3), which is repressive to gene activity, while the TrxG/MLL complex catalyzes the methylation of histone H3 at lysine 4 (H3K4me3), which acts as an activating mark^19,20^.

An orthologous human FENDRR was also identified^21^. The human FENDRR gene is 3,099 bp in length, located at chr3q13.31, and consists of four exons. Xu et al. has shown that FENDRR is dramatically downregulated in gastric cancers and that the low expression of FENDRR is associated with invasion depth, tumor stage, lymphatic metastasis and patient survival time. Moreover, upregulation of FENDRR suppresses gastric cancer cell migration and invasion in vitro by targeting FN1 and MMP2/MMP9^22^. However, the role of FENDRR in macrophage polarization is unknown.

In this study, we found that FENDRR had a low expression level in human monocyte-derived macrophages and was highly induced in IFNγ-stimulated M1 macrophages. Overexpression of FENDRR enhanced M1 macrophage polarization, while knockdown of FENDRR had an opposite effect, suggesting a role of FENDRR in M1 macrophage polarization.

## Materials and methods

### IFNγ- and IL4-induced macrophage polarization

THP-1 cells (TIB-202, ATCC, Manassas, VA, USA) were grown in RPMI 1,640 medium containing 0.05 mM 2-mercaptoethanol (Sigma-Aldrich, Saint Louis, MO, USA) and 10% heat-inactivated fetal bovine serum (FBS, Atlanta Biologicals Inc., Flowery Branch, GA, USA). THP-1 cells (2 × 10^6^/well) were seeded in a 6-well plate and differentiated into macrophages by treatment with 320 nM phorbol-12-myristate-13-acetate (PMA) (Promega Corporation, Madison, WI, USA) overnight. The PMA-activated THP-1 cells (THP-1 macrophages) were treated with either 20 ng/mL human recombinant IFNγ (PeproTech, Rocky Hill, NJ, USA) for M1 polarization or 20 ng/mL human recombinant IL4 (PeproTech, Rocky Hill, NJ, USA) for M2 polarization. Non-polarized PMA-activated THP-1 cells were used as a control. After 4, 8, 24 and 48 h polarization, the adherent cells were harvested and used for further analysis.

### RNA isolation and DNase I treatment

Total RNA was extracted using TriReagent (Molecular Research Center Inc., Cincinnati, OH, USA), according to the manufacturer’s instructions. RNA concentration was measured using NanoDrop ND-100. Five µg of total RNA was treated with DNase I (Thermo Fisher Scientific, Waltham, MA, USA), according to manufacturer’s protocol, followed by phenol chloroform RNA purification.

### Quantitative real-time polymerase chain reaction (qPCR)

cDNA synthesis was performed using 1 µg DNase I-treated RNA and 200 U/µL MMLV (Thermo Fisher Scientific). Real-Time PCR reaction was performed with 5 times-diluted cDNA and specific primers (Table 1) using qPCR Master Mix Plus for SYBR green (Eurogentec, AnaSpec, Fremont, CA, USA) on an Applied Biosystems 7,500 fast Real Time PCR instrument. Relative gene expression of lncRNA and mRNA was analyzed by the 2^(−ΔΔCT)^ method, using GAPDH as a reference gene.

**Table 1:** Human and mouse qPCR primers.

Genes	Primer sequences	
**qPCR human primers**		
GAPDH	Forward	GAAGGTGAAGGTCGGATG
	Reverse	GAAGATGGTGATGGGATT
FENDRR	Forward	GCGCACAGACCCAGGATTT
	Reverse	ACACGGGCAGAGCTGGTTT
TNFα	Forward	GCAGGTCTACTTTGGGATCATTG
	Reverse	GCGTTTGGGAAGGTTGGA
IL1β	Forward	CCACAGACCTTCCAGGAGAAT
	Reverse	GTGCAGTTCAGTGATCGTACAGG
IL6	Forward	AGACAGCCACTCACCTCTTCAG
	Reverse	TTCTGCCAGTGCCTCTTTGCTG
IL10	Forward	TCCAGTGTCTCGGAGGGATT
	Reverse	TGGCCACAGCTTTCAAGAATG
CCL22	Forward	ATTACGTCCGTTACCGTCTGC
	Reverse	TCCCTGAAGGTTAGCAACACC
**qPCR mouse primers**		
GAPDH	Forward	CTCGTCCCGTAGACAAAATGGT
	Reverse	TGATGGCAACAATCTCCACTTT
TNFα	Forward	GGTGCCTATGTCTCAGCCTCTT
	Reverse	GCCATAGAACTGATGAGAGGGAG
IL1β	Forward	GAAATGCCACCTTTTGACAGTG
	Reverse	CTGGATGCTCTCATCAGGACA
CXCL10	Forward	ATCATCCCTGCGAGCCTATCCT
	Reverse	GACCTTTTTTGGCTAAACGCTTTC
ARG1	Forward	CAGAAGAATGGAAGAGTCAG
	Reverse	CAGAT ATGCAGGGAGTCACC
FIZZ1	Forward	CCAATCCAGCTAACTATCCCTCC
	Reverse	ACCCAGTAGCAGTCATCCCA
**Primers for the construction of human FENDRR shRNA vector**		
FENDRR-shRNA	Forward	GATCCGATTTGCCAGCAACTGCATCATTCAAGAGATGATGCAGTTGCTGGCAAATCTTTTTG
FENDRR-shRNA	Reverse	AATTCAAAAAGATTTGCCAGCAACTGCATCATCTCTTGAATGATGCAGTTGCTGGCAAATCG

### Lentiviral FENDRR expression vector

FENDRR (transcript variant 3, GenBank# MK522493.1) was amplified by PCR using cDNA from human lung tissue and inserted into a lentiviral vector at the XhoI and EcoRI sites as described^23,24^. The control vector was constructed with a random genomic DNA insert that did not contain any known lncRNAs or mRNAs. All the inserts in the plasmid constructs were confirmed by DNA sequencing. Lentiviruses were produced using the Lenti-X™ HTX Packaging vectors (Clontech, Mountain View, CA) in HEK 293T cells. The virus titer was determined by infecting HEK 293T cells with a series of dilutions of the viral stock and counting the virus-infected green fluorescent protein (GFP)-positive cells.

### FENDRR overexpression in non-activated suspension THP-1 cells (spinoculation of suspension cells)

Non-activated THP-1 cells (2 × 10^6^) were resuspended into 2 mL of complete culture media containing 8 µg/mL polybrene (Sigma-Aldrich). FENDRR or control lentivirus was added at a multiplicity of infection (MOI) of 50 and incubated for 30 min at room temperature. After a brief mix by pipetting, cells were spun at 800×*g* for 2 h at 32 °C. Lentivirus-infected cells were seeded at 1 × 10^6^ cells/well in a 6-well plate and incubated for 24 h at 37 °C. The media was replaced the next day with fresh complete culture media, and the cells were incubated for another 72 h.

### FENDRR overexpression in PMA-activated THP-1 macrophages

THP-1 cells (2 × 10^6^/well) were seeded in a 6-well plate and activated overnight with 320 nM PMA. Media was removed and 2 mL of fresh RPMI 1,640 media containing 8 µg/ mL polybrene. FENDRR or control lentivirus (MOI, 50) was added to the well. After a 24-h incubation at 37 °C, the media was replaced with fresh complete culture media, and the cells were incubated for another 72 h.

### FENDRR overexpression in mouse bone marrow-derived macrophages

Bone marrow-derived macrophages (BMDM) were isolated from 8 to 10 weeks old C57Bl/6 mice according to Inés Pineda-Torra et al.^25^. Briefly, bone marrow was flushed out with cold Phosphate Buffered Saline (PBS) from tibiae and femurs of one mouse, strained through a 70 µm cell strainer (BD Biosciences, Flanklin Lakes, NJ, USA) and centrifuged at 300×*g* for 5 min. Cell pellet was then resuspended in the warm differentiation medium containing DMEM, 15% L929 conditioned medium, 10% FBS and 1% penicillin/streptomycin. The cells were plated on a non-treated 150 mm cell culture dish (Corning, New York, NY, USA) and incubated in a humidified incubator with 5% CO_2_ at 37 °C. Macrophages were fully differentiated after 6 days. FENDRR was overexpressed in fully differentiated BMDM using FENDRR or control lentivirus (MOI, 50) in DMEM containing 8 µg/mL polybrene, 10% FBS and 1% penicillin/streptomycin. After a 24-h incubation at 37 °C, the medium was replaced with fresh DMEM media containing 10% FBS and 1% penicillin/streptomycin and the cells were incubated for another 24 h. Real-time PCR was used to determine FENDRR and cell marker expression in BMDM.

### FENDRR shRNA

shRNAs were designed by the BLOCK-iT™ RNAi Designer software from Invitrogen (Grand Island, NY, USA). The FENDRR shRNA was inserted into the pSIH-H1 vector (System Biosciences, Mountain View, CA, USA), which utilizes the H1 promoter to drive shRNA expression. A control vector containing scrambled shRNA was purchased from System Biosciences. The primers used for the construction of FENDRR shRNA are listed in Table 1. The shRNA in the plasmid was confirmed by DNA sequencing. Lentiviruses were produced and titrated as described above. Cells were infected with a lentivirus expressing shRNA targeting FENDRR or a control virus at an MOI of 50 for 48 h. Real-time PCR was then used to determine FENDRR level.

### Cytokine protein levels

IL1β and TNFα protein levels were measured in the cell culture supernatant by enzyme linked immunosorbent assay (R&D Inc., Minneapolis, MN, USA–Quantikine ELISA), according to the manufacturer’s instructions.

### Western blotting analysis of phosphorylated STAT1

Macrophages were lysed in lysis buffer (T-PER, Thermo Fisher Scientific) containing a protease and phosphatase inhibitor cocktail (Thermo Fisher Scientific) for 30 min on ice. Cell debris was removed by centrifugation at 15,000×*g* for 10 min at 4 °C. Protein concentration in the cell lysate was determined using a BioRad protein assay kit (BioRad, Hercules, California, USA). The proteins in each sample (10 µg) were separated by 10% SDS-PAGE, and subsequently transferred onto a nitrocellulose membrane using the BioRad Turbo Trans system. After blocking with 5% skim milk for 1 h in TTBS (20 mM Tris, 150 mM NaCl, and 0.05% Tween 20, pH 7.5), membranes were incubated with primary antibodies, anti-phospho STAT1-Y701 (1:1,000 dilution, Cell Signaling, Beverly, MA, USA), or mouse anti-human β-actin (1:3,000 dilution, Thermo Fisher Scientific) overnight and then for 1 h with the respective secondary antibodies (1:3,000 dilution, goat anti-rabbit and goat anti-mouse HRP conjugated, Jackson Immuno Research, USA). Blots were developed using Super Signal West Pico (Thermo Fisher Scientific), and signals were detected with Amersham Imager 600. Intensity of the bands was quantified by ImageJ densitometry with β-actin as a loading control.

### Statistical analysis

All experiments were repeated three times. Data were shown as the mean ± standard deviation (SD). One-way and two-way ANOVA, followed by a Tukey’s post hoc test were performed for multiple group comparisons using GraphPad Prism software. *P* < 0.05 was considered statistically significant.

## Results

### FENDRR expression in polarized macrophages

**Figure 1:**
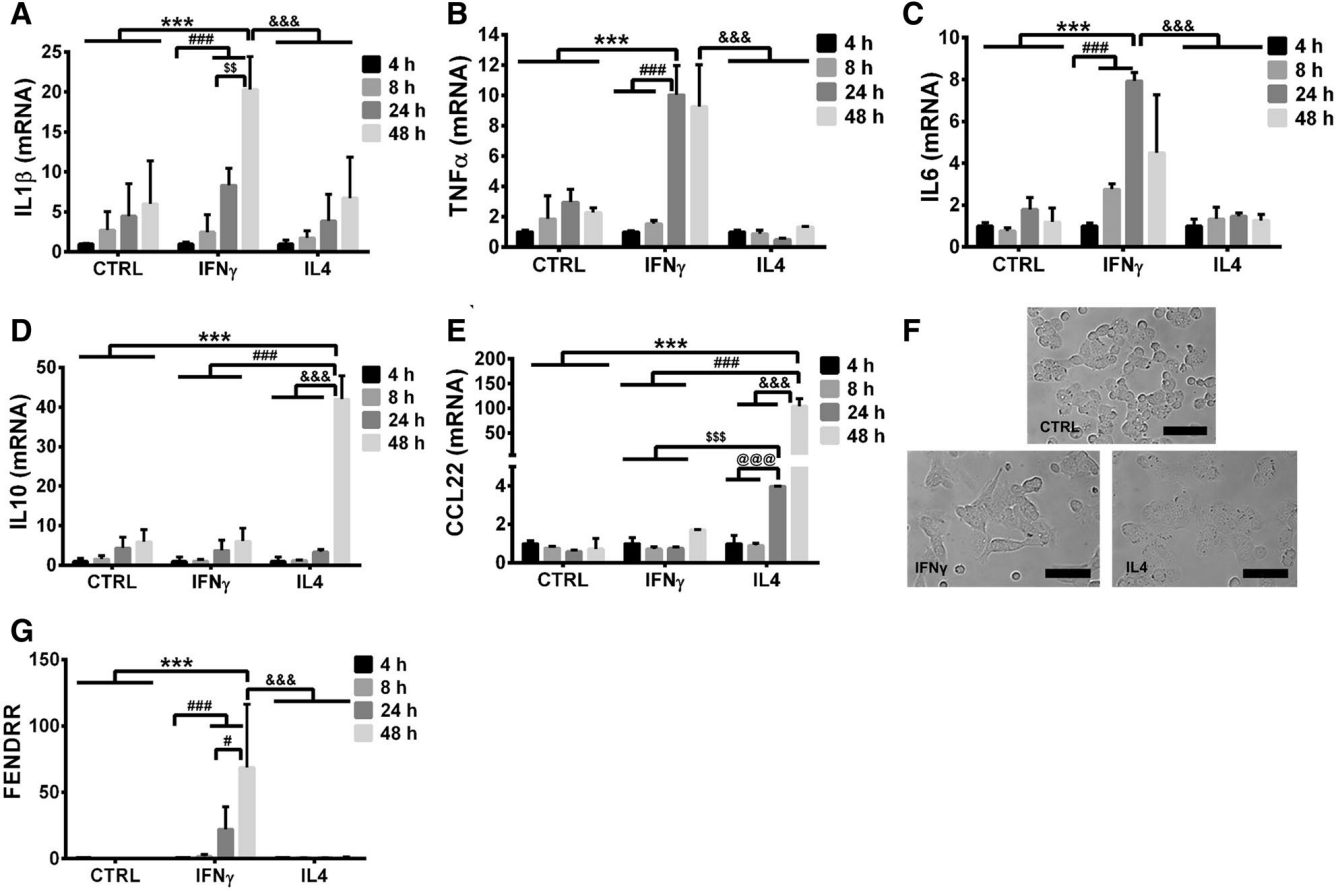
IFNγ-polarized M1 macrophages have an increased FENDRR expression. (**A**–**C**) show increased mRNA levels of M1 markers, IL1β, TNFα and IL6 in IFNγ-polarized THP-1 macrophages. (**D**,**E**) shows increased mRNA levels of M2 markers, IL10 and CCL22 in IL4-polarized THP1 macrophages. (**F**) shows bright field images of PMA-activated THP-1 macrophages untreated (top panel) or treated with 20 ng/mL IFN-γ (bottom left panel) or IL-4 (bottom right panel), scale bar—50 µm. M1 phenotype is associated with an elongated cell shape, while M2 phenotype has a flattened and rounded shape. (**G**) Shows that lncRNA FENDRR was expressed 80 times higher in M1 (IFNγ) than that in M2 (IL4) polarized macrophages. The results were normalized to GAPDH and expressed as a fold change to 4 h. Data are presented as the mean ± SD. n = 3. ***P < 0.001, ^#^P < 0.05, ^###^P < 0.001, ^&&&^P < 0.001, ^$$^P < 0.01, ^$$$^P < 0.001, ^@@@^P < 0.001. Two-way ANOVA, followed by Tukey’s post hoc test. CTRL: control cells.

M1 and M2 macrophages were generated by treating THP-1 cells with PMA and polarizing the cells with IFNγ and IL4. The PMA-treated THP-1 served as controls. IFNγ increased the mRNA expression of the M1 marker, TNFα and IL1β at 48 h and IL6 at 24 h compared to controls at the same time points (Fig. 1A–C). On the other hand, IL4 increased the mRNA expression of the M2 markers, IL10 at 48 h and CCL22 at 24 h and 48 h (Fig. 1D,E). We also observed that the THP-1 macrophages that polarized toward different phenotypes exhibited dramatic changes in cell shape: IL4-induced M2 cells had a rounded shape with elongated filopodia, while IFNγ-induced M1 cells adopted an elongated, spindle-shaped cell morphology (Fig. 1F). These results confirmed the M1 and M2 polarization models.

We next examined the expression of FENDRR in IFNγ- and IL4-polarized THP-1 macrophages. Our data showed that IFNγ treatment of THP-1 macrophages significantly increased FENDRR expression at 24 and 48 h (Fig. 1G). However, IL-4 had no effects on FENDRR expression. These results suggest that FENDRR may play a role in IFNγ-induced M1 macrophage polarization.

### Effect of FENDRR overexpression on M1 macrophage polarization

**Figure 2:**
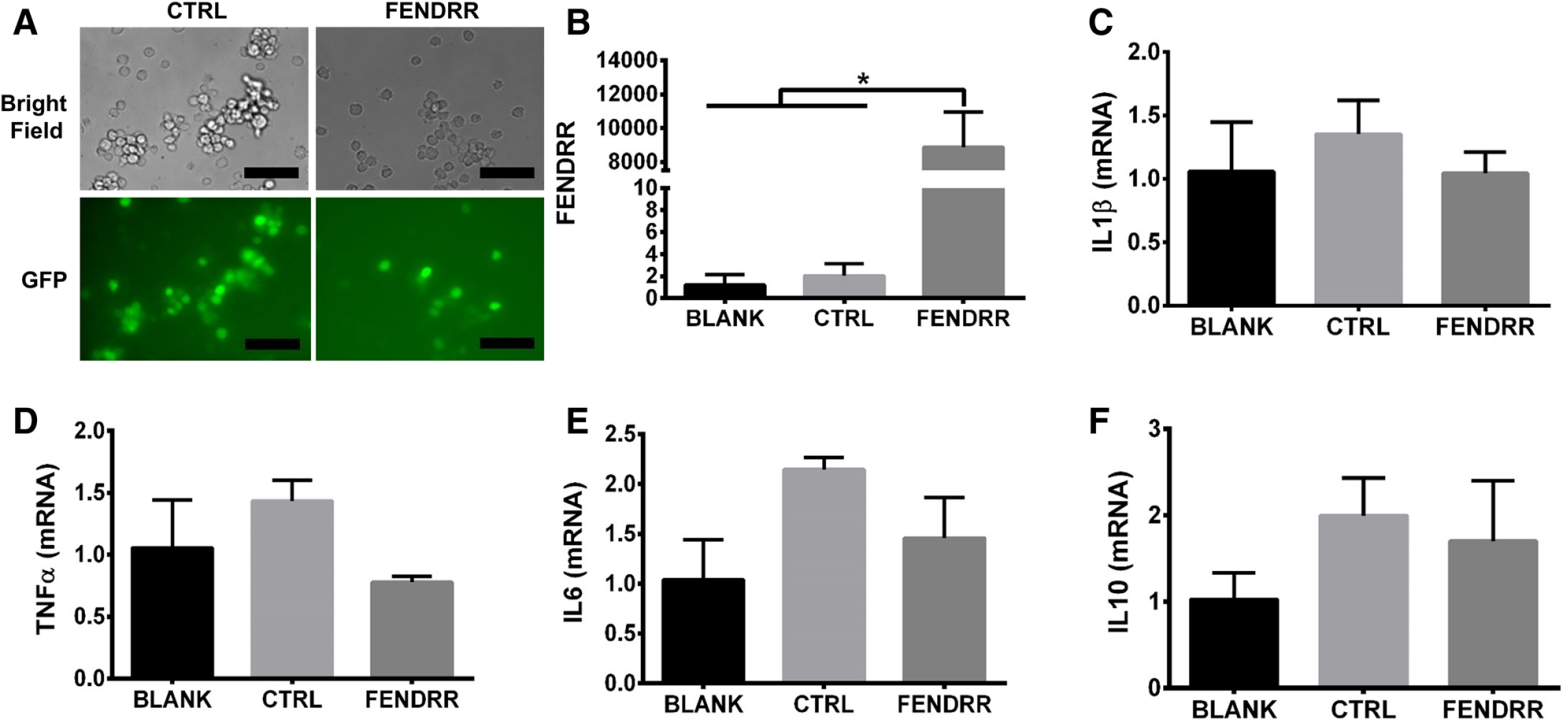
M1 and M2 macrophage marker expression was not affected by FENDRR overexpression in human monocytes. (**A**) Bright field and GFP fluorescence of THP-1 monocytes 96 h after control or FENDRR lentivirus infection. Scale bar—50 µm. (**B**) FENDRR expression in THP-1 monocytes 96 h post spinoculation.(**C**–**F**) The mRNA expression of IL1, TNFα, IL6, and IL10 shows no changes in THP-1 monocytes 96 h after FENDRR overexpression. The results were normalized to GAPDH and expressed as a fold change to blank. Data are presented as the fold change mean ± SD. n = 3. *P < 0.05. One-way ANOVA, followed by Tukey’s post hoc test. BLANK: medium, CTRL: control lentivirus, FENDRR: FENDRR lentivirus.

Because FENDRR expression was significantly increased by IFNγ treatment, we wanted to know if FENDRR overexpression can induce M1 macrophage phenotype. We first determined whether FENDRR can directly convert THP1 monocytes to M1 macrophages. THP1 cells were infected with a FENDRR lentivirus by spinoculation. GPF images showed a high infection efficiency (Fig. 2A). FENDRR overexpression was confirmed in the lentivirus-treated THP1 cells compared to virus control-infected or blank cells (Fig. 2B). There were no significant differences in the expression of M1 macrophage markers, TNFα, IL1β, and IL6, and M2 macrophage marker, IL10 between the FENDRR overexpressing and control groups (Fig. 2C–F), suggesting that FENDRR does not induce M1 and M2 phenotypes in non-activated monocytes.

**Figure 3:**
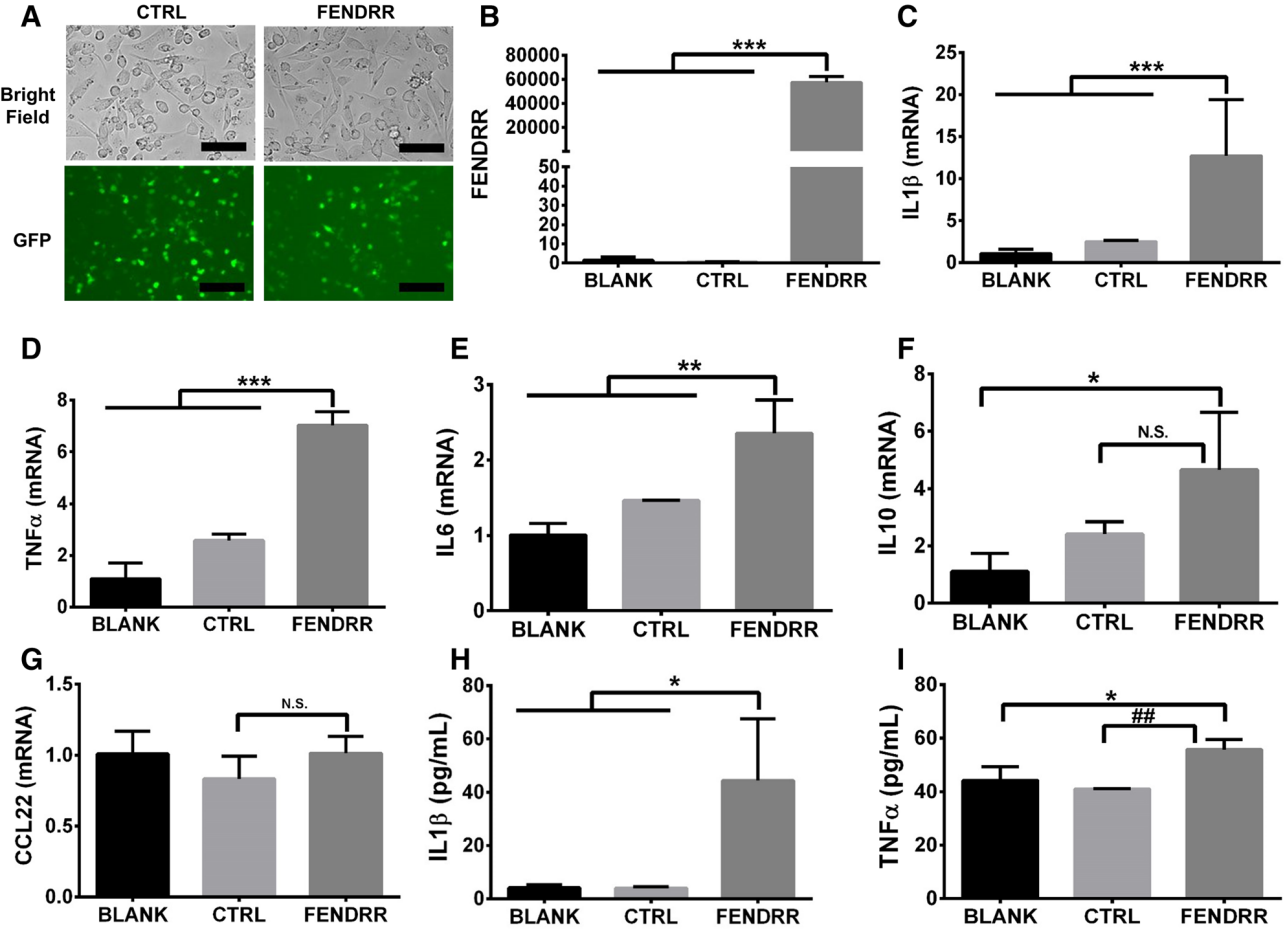
FENDRR overexpression induces M1 marker expression in PMA-activated THP-1 macrophages. (**A**) Bright field and GFP fluorescence of THP-1 macrophages 96 h after control or FENDRR lentivirus infection. Scale bar—50 µm. (**B**–**G**) Increased FENDRR level in the PMA-activated THP-1 macrophages 96 h after lentivirus infection is associated with increased mRNA expression of M1 markers, IL1β, TNFα and IL6, but not M2 markers, IL10 and CCL22. (**H**,**I**) Increased production of IL1β and TNFα in the supernatant from PMA-activated THP-1 macrophages overexpressing FENDRR, as quantified by ELISA. The results are presented as the mean ± SD. n = 3. *P < 0.05, **P < 0.01, ***P < 0.001, ^##^P < 0.01, NS: not significant. One-way ANOVA, followed by Tukey’s post hoc test. BLANK: medium, CTRL: control lentivirus, FENDRR: FENDRR lentivirus.

We then determined whether overexpression of FENDRR in the PMA-activated THP-1 macrophages can induce the M1 macrophage phenotype. The high infection efficiency and overexpression of FENDRR in the PMA-activated THP1 macrophages are shown in Fig. 3A,B. FENDRR overexpression increased the mRNA expression of M1 markers, IL1β, TNFα and IL6 and IL1β, TNFα protein levels released into the culture media, but had no significant effects on the mRNA expression of M2 markers, IL10 and CCL22 compared to the virus control (Fig. 3C–I), suggesting that FENDRR induces M1 but not M2 polarization. FENDRR appears to increase the IL10 mRNA level compared to blank control. This is likely due to the effects of the lentiviral system that we used to overexpress FENDRR since the control virus also increased IL10 expression and there was no significant difference in IL10 levels between the virus control and FENDRR group.

**Figure 4:**
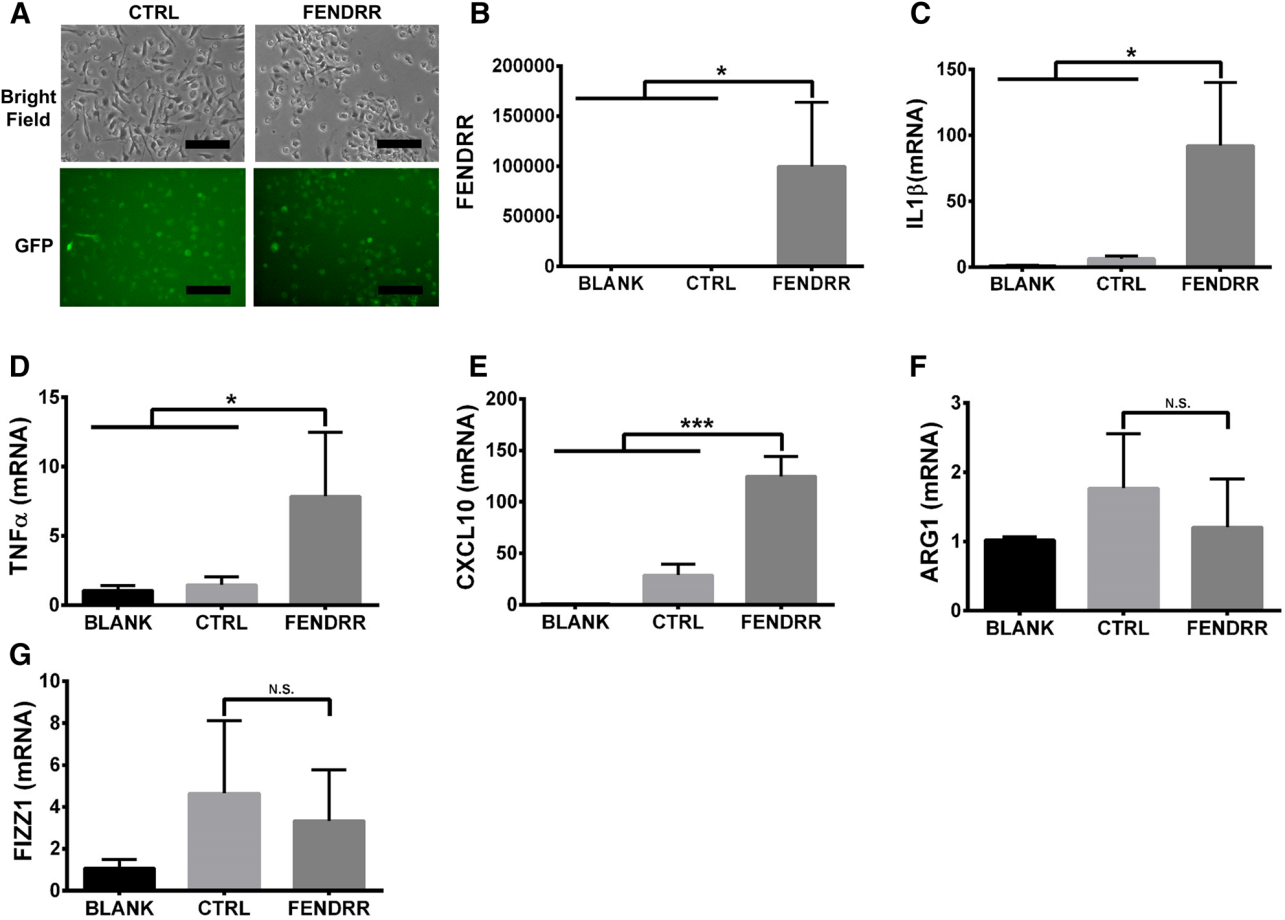
FENDRR overexpression induces M1 marker expression in bone marrow-derived macrophages. (**A**) Bright field and GFP fluorescence of mouse BMDM macrophages 48 h after control or FENDRR lentivirus infection. Scale bar—50 µm. (**B**–**G**) Increased FENDRR level in BMDM 48 h after lentivirus infection is associated with increased mRNA expression of M1 markers, IL1β, TNFα and CXCL10, but not M2 markers, ARG1 and FIZZ1. The results were normalized to GAPDH and expressed as a fold change to blank. Data are presented as the mean ± SD. n = 3. *P < 0.05, **P < 0.01, ***P < 0.001, NS: not significant. One-way ANOVA, followed by Tukey’s post hoc test. BLANK: medium, CTRL: control lentivirus, FENDRR: FENDRR lentivirus.

Since THP-1 is derived from acute monocytic leukemia^26,27^, we examined whether FENDRR also induced M1 polarization in primary mouse bone marrow-derived macrophages (BMDM). We confirmed the high infection efficiency and overexpression of FENDRR in BMDM using the lentiviral expression system (Fig. 4A,B). Similar to PMA-activated THP-1 macrophages, we observed that FENDRR overexpression in BMDM increased the mRNA expression of mouse M1 markers, IL1β, TNFα and CXCL10 (Fig. 4C–E) and did not significantly affect the mRNA expression of mouse M2 markers, arginase 1 (ARG1) and found in inflammatory zone 1 (FIZZ1) (Fig. 4F,G).

**Figure 5:**
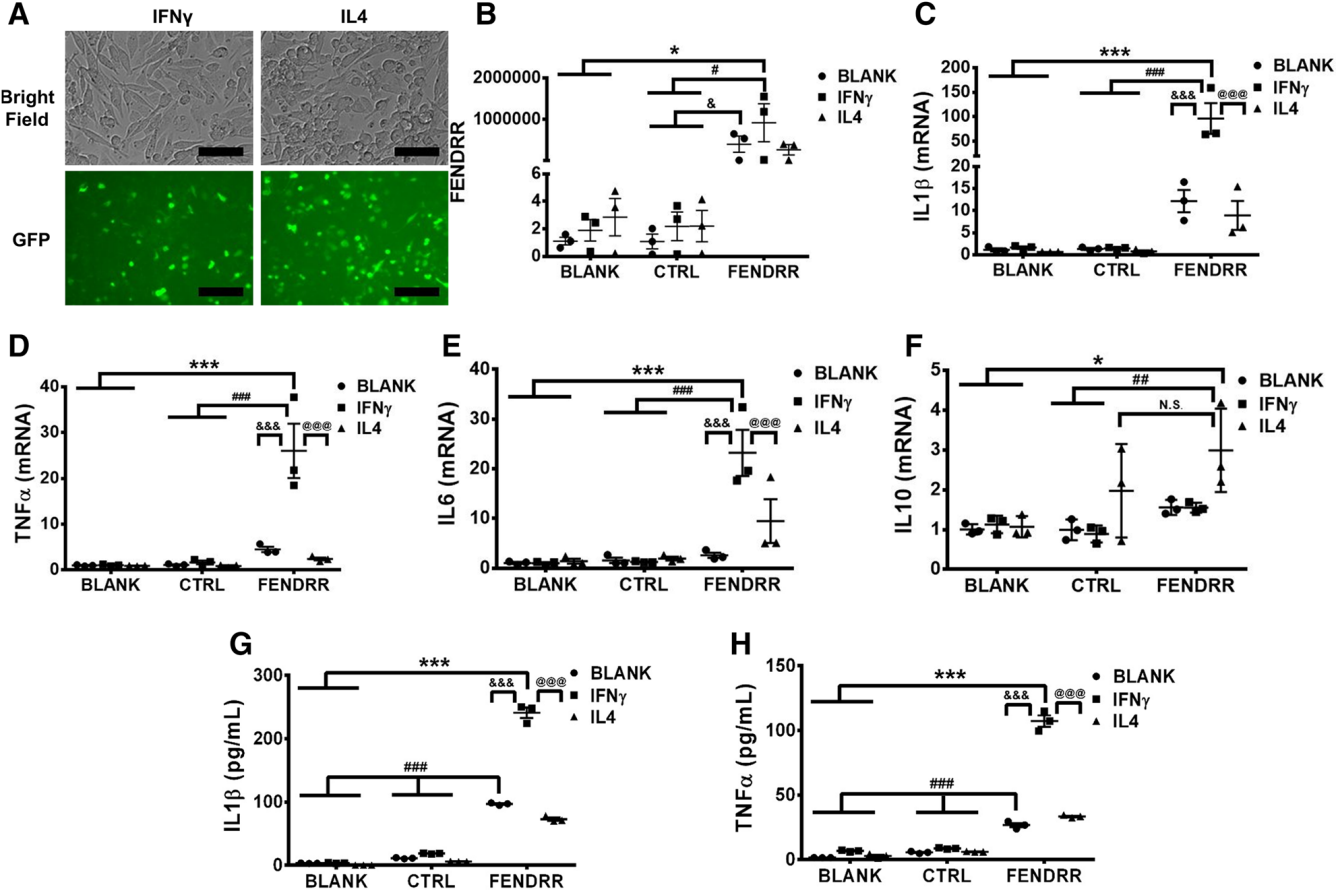
FENDRR and IFNγ synergistically increase M1 marker expression in PMA-activated THP-1 macrophages. (**A**) Bright field and GFP fluorescence of PMA-activated THP-1 macrophages 48 h after lentiviral FENDRR infection, followed by 48 h IFNγ or IL4 polarization. Scale bar—50 µm. (**B**–**F**) FENDRR level and the mRNA expression of M1 markers, IL1β, TNFα, and IL6 and M2 markers, IL10 in IFNγ- or IL4-polarized macrophages. The results were normalized to GAPDH and expressed as a fold change to blank. (**G**,**H**) The production of IL1β and TNFα in the supernatant of IFNγ- or IL4-polarized macrophages as determined by ELISA. Data are presented as the mean ± SD. n = 3. *P < 0.05, ***P < 0.001, ^#^P < 0.05, ^##^P < 0.01, ^###^P < 0.001, ^&^P < 0.05, ^&&&^P < 0.001, ^@@@^P < 0.001. Two-way ANOVA, followed by Tukey’s post hoc test. BLANK: medium, CTRL: control lentivirus, FENDRR: FENDRR lentivirus.

Finally, we determined whether FENDRR can enhance IFNγ-induced M1 polarization. The PMA-activated THP1 cells were infected with a FENDRR lentivirus for 48 h and then treated with IFNγ or IL4 for another 48 h. Once again, infection efficiency and overexpression of FENDRR was confirmed (Fig. 5A,B). FENDRR overexpression further increased IFNγ-induced mRNA expression of IL1β, TNFα and IL6 but had little effect on IL10 expression compared to control virus (Fig. 5C–F). Using ELISA, we also observed that FENDRR increased IFNγ-induced IL1β and TNFα proteins released into the media (Fig. 5G,H). Our data suggest that IFNγ and FENDRR had a synergic effect on M1 polarization.

### Knockdown of FENDRR inhibits IFNγ-induced M1 macrophage polarization

**Figure 6:**
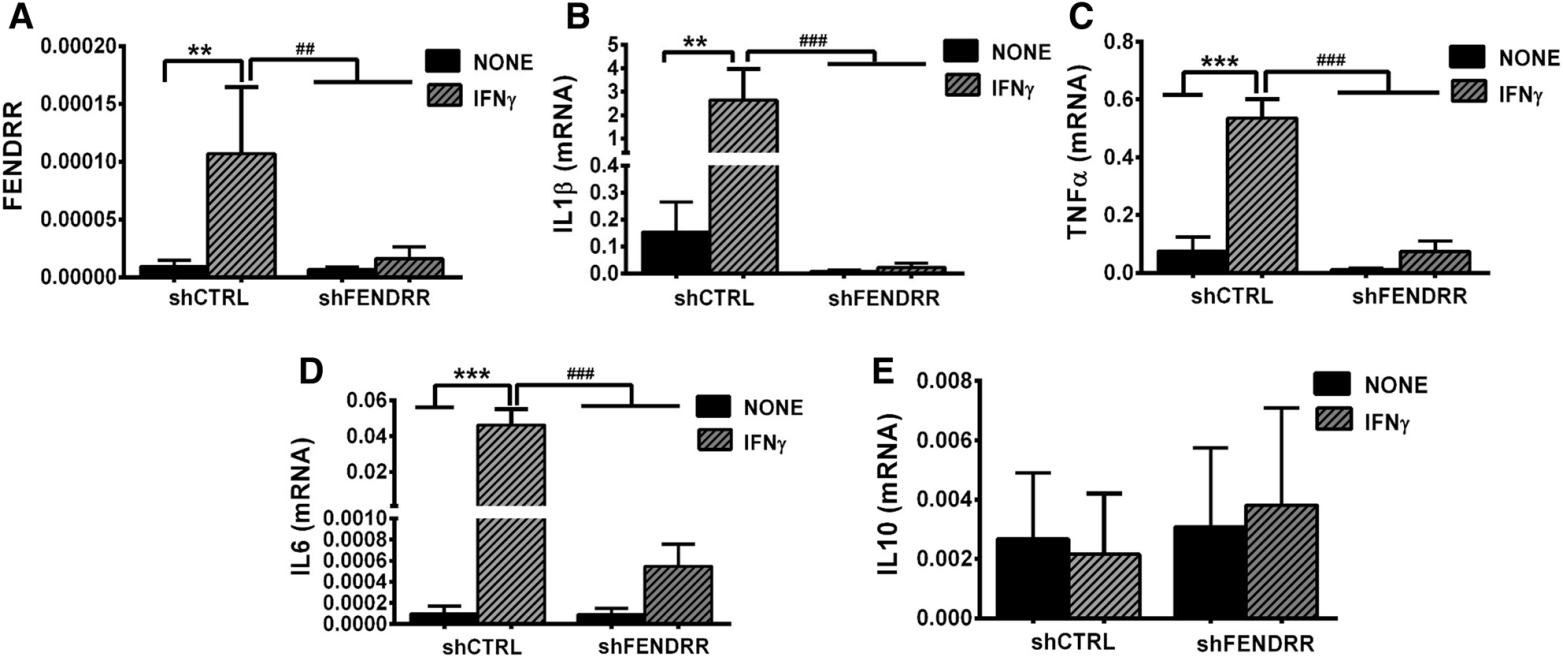
Knockdown of FENDRR suppresses IFNγ-induced M1 phenotype. (**A**) FENDRR level in IFNγ-polarized macrophages was reduced by shRNA FENDRR. (**B**–**E**) IFNγ-induced M1 marker expression (IL1β**,** TNFα, and IL6**),** but not M2 marker expression (IL10) was suppressed by FENDRR knockdown. The results were normalized to GAPDH. Data are presented as the mean ± SD. n = 3. **P < 0.01, ***P < 0.001, ^##^P < 0.01, ^###^P < 0.001. One-way ANOVA, followed by Tukey’s post hoc test. shCTRL: control shRNA lentivirus, shFENDRR: FENDRR shRNA lentivirus.

To further confirm the effects of FENDRR on M1 macrophage polarization, we knocked down FENDRR by infecting the PMA-activated THP-1 cells with a lentivirus containing shRNA targeting FENDRR, followed by IFNγ-induced M1 polarization. FENDRR expression was effectively reduced by the shRNA (Fig. 6A). The reduction of FENDRR blocked IFNγ-induced IL1β, TNFα and IL6 mRNA expression but had no effects on IL-10 expression (Fig. 6B–E), further supporting that FENDRR promotes M1 macrophage polarization.

### FENDRR acts via STAT1 signaling

**Figure 7:**
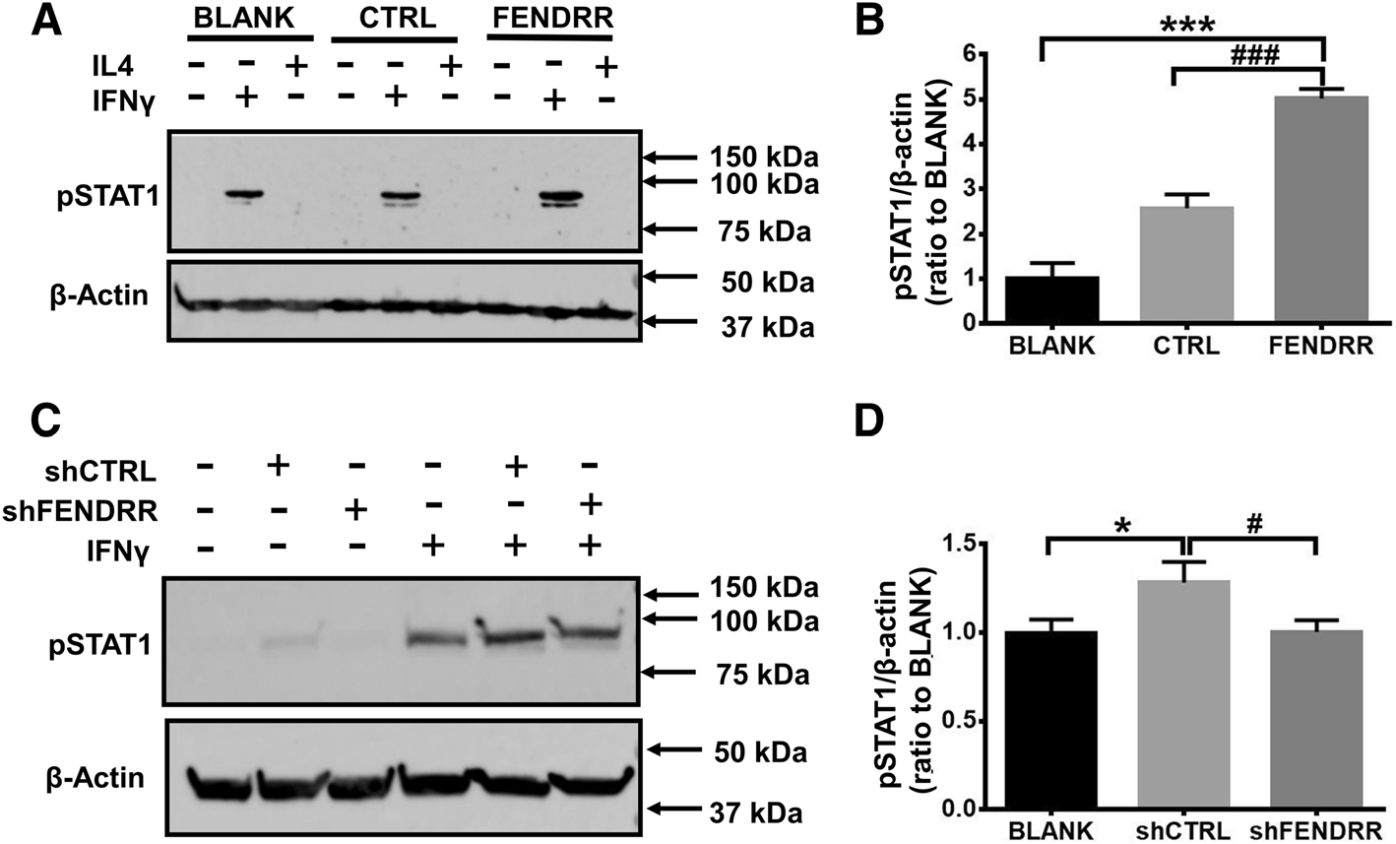
Effect of FENDRR overexpression and knock-down on STAT1 phosphorylation. (**A**,**B**) PMA-activated THP1 macrophages overexpressing FENDRR were polarized with IFNγ for 48 h. (**C**,**D**) FENDRR was silenced using shRNA FENDRR lentivirus after 48 h IFNγ polarization. Phosphorylated STAT1 was detected by western blot with specific antibodies and quantitated. The results are presented as the mean ± SD. n = 3. *P < 0.05, ***P < 0.001, ^#^P < 0.05, ^###^P < 0.001. One-way ANOVA, followed by Tukey’s post hoc test. BLANK: medium, CTRL: control lentivirus virus, FENDRR: FENDRR lentivirus. shCTRL: control shRNA lentivirus, shFENDRR: FENDRR shRNA lentivirus.

STAT1 is the primary mediator for IFNγ signaling^28^. To gain insight into the underlying mechanism of FENDRR-mediated M1 polarization, we examined whether FENDRR influences the STAT1 phosphorylation. The results showed that IFNγ increased the phosphorylation of STAT1, and FENDRR overexpression further increased STAT1 phosphorylation, as demonstrated by western blot using anti-phosphoSTAT1 (Tyr701) (Fig. 7A,B). On the other hand, silencing FENDRR reduced STAT1 phosphorylation (Fig. 7C,D). These results suggest that FENDRR-induced M1 polarization functions via STAT1.

## Discussion

The highly dynamic phenotype and function of macrophages can be shaped by different environmental signals^29^. Progress has been made in defining the mechanisms underlying macrophage polarization^30^. However, the role of lncRNAs in macrophage polarization is less known. In this study, we identified lncRNA FENDRR as a positive regulator of M1 macrophage polarization.

Among the multiple factors involved in the regulation of macrophage polarization, noncoding RNAs have been recognized as important regulatory molecules. MicroRNAs (miRNAs) have emerged as positive or negative regulators of M1 polarization^31^. For example, miR-21, miR-29a and let-7b were found to induce TNFα and IL6 in microglia and macrophages by binding TLR7 (TLR8 in humans)^32,33^, suggesting that they may be involved in M1 macrophage polarization. These miRNAs function as a TLR7 ligand as they have a similar GU content and length as the known TLR7 ligand, ssRNA40. Several miRNAs have been shown to regulate macrophage polarization by modulating transcription factors and signaling pathways involved in M1 and M2 polarization^34^. miR-125b increases macrophage responsiveness to IFNγ by targeting the transcription factor, IRF4 that inhibits NF-kB activity, thereby promoting M1 phenotype macrophages^35^. miR-27 and miR-130 promote pro-inflammatory macrophage polarization by interacting with PPARγ, while miR-155 and miR-21 enhanced pro-inflammatory responses by activating STAT1 and STAT3 pathways^36–39^. Most recently, miR-216a was found to enhance M1 and suppress M2 macrophage polarization by regulating telomerase activity through SMAD3/NF-kB pathway^40^.

**Figure 8:**
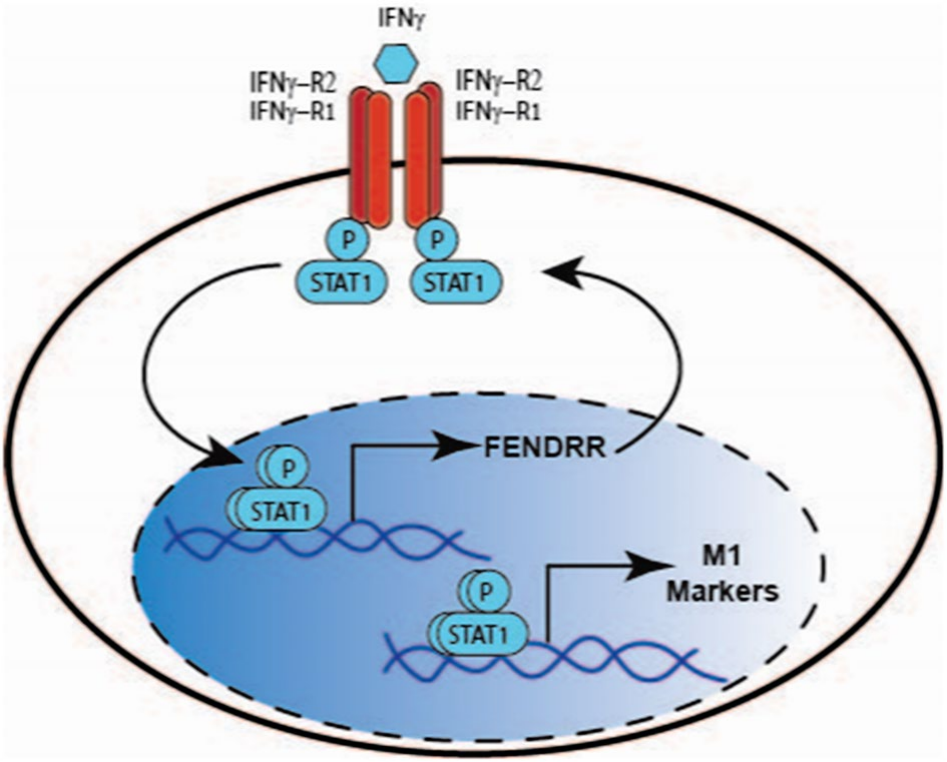
A model for FENDRR-mediated M1 macrophage polarization. Upon IFNγ binding to IFNγ receptors, STAT1 is phosphorylated and translocated to the nucleus, which activates transcription of lncRNA FENDRR and M1 polarization markers. FENDRR further enhances STAT1 phosphorylation, forming a forward feedback loop.

Many lncRNAs are expressed in a cell type- and state-specific manner, and their expression is tightly regulated by various cellular signals^11,41^. Our current study shows that IFNγ, but not IL4, induces FENDRR expression in human macrophages. Hundreds of intergenic lncRNAs are modulated by JAK-STAT signaling in T helper cells^12^. LncRNA BANCR expression is induced in human retinal pigment epithelial cells by IFN-γ, but not TNFα or IL1β and a JAK inhibitor blocks this effect^42^. Using the PROMO online software, we identified two STAT3 binding sites in the 5 kb FENDRR promoter, suggesting that IFNγ may regulate FENDRR expression in macrophage through STAT1/3 heterodimers. FENDRR also enhances IFNγ-mediated STAT1 phosphorylation, indicating a forward feedback regualtion of FENDRR expression by IFNγ (Fig. 8).

Compared to miRNAs, much less are known regarding the roles of lncRNAs in macrophage polarization. LncRNA THRIL mediates the pro-inflammatory response of PMA-activated THP1 macrophages by interacting with heterogeneous nuclear ribonucleoproteins^43^. LncRNA GAS5 promotes M1 polarization through sponging miR-455-5p^44^. LncRNA Malat1 enhances M1 macrophage polarization, but inhibits M2 phenotype. Myeloid specific knockout of Malat1 in mice has a reduced LPS-induced lung inflammation, but an increased lung fibrosis caused by bleomycin^45^. lncRNA-MMP2 is upregulated in M2 polarized macrophages and is required for M2 polarization through STAT6 activation. However, the mechanism of lncRNA-MM2P-mediated STAT6 phosphorylation remains unknown^46^.

Our current studies uncovered lncRNA FENDRR as a new positive regulator of M1 macrophage polarization. It has been long appreciated that IFNγ-induced signaling typically augments M1 macrophage polarization through a STAT1-dependent mechanism. IFNγ, the sole member of the type II interferon family, acts via binding the IFNγ receptor and signaling through the JAK/STAT pathway, leading to STAT1 phosphorylation, nuclear translocation and induction of transcription of STAT1-regulated genes^47^. It has been reported that lncRNA MacORIS inhibits JAK2 and STAT1 phosphorylation^48^. Our data shows that overexpression of FENDRR increased and knock-down of FENDRR reduced IFNγ-mediated phosphorylation of STAT1, suggesting that enhancement of M1 polarization by FENDRR occur via the STAT1-dependent pathway.

How FENDRR regulates STAT1 signaling and M1 macrophage polarization remains to be determined. Based on literature, we speculated several possibilities. The first possibility may involve epigenetic regualtion. Fendrr has been shown to interact with both PRC2 and TrxG/Mll complexes via dsDNA/RNA triplex formation at target regulatory elements and to increase PRC2 occupancy at these sites, in addition to enhanced trimethylation of histone 3 lysine 4 (H3K4me3) and histone 3 lysine 27 (H3K27me3) at the promoter site of the target genes^49^. This mechanism is consistent with two previous findings: (a) histone methyltransferase MLL is upregulated in M1 macrophages, which increases H3K4me3 at the promoter of pro-inflammatory cytokine CXCL10^50^. (b) PRC2-mediated EZH2-dependent H3K27 methylation suppresses several anti-inflammatory genes such as MERTK, PPARG and RANK in IFNγ-polarized macrophages^51^.

The second possibility is that FENDRR may function as a miRNA sponge. FENDRR has been recently shown to act as a molecular sponge for miRNA-18a-5p and miR-126 in prostate, gallbladder cancer and human brain microvascular endothelial cells^52–54^.

The third possibility is that FENDRR may promote M1 macrophage polarization through iron-mediated repression of STAT1 signaling. Iron metabolism genes are differentially expressed in M1 and M2 macrophages. Compared to M2 macrophages, M1 macrophages have a higher expression of ferritin (iron storage) and a lower expression of ferroportin (iron export), transferrin receptor (iron import) and iron regulatory protein 1 and 2^55,56^. Iron suppresses M1 polarization in Raw 264.7 macrophages, mouse bone marrow-derived macrophages and THP-1 monocyte-derived macrophages^57–59^ and promotes M2 polarization in THP-1 monocyte-derived macrophages^59,60^. However, one study reported opposite results showing that iron increases M1 macrophage markers, but inhibits IL-4-induced M2 macrophage markers in mouse bone marrow-derived macrophages^61^. Iron decreases STAT1 phosphorylation in IFNγ-treated RAW 246.7 macrophages^57^, which is consistent with the iron-mediated inhibition of M1 macrophage polarization. We have recently shown that FENDRR reduces iron levels in lung fibroblasts by interacting with iron regulatory protein-1 to inhibit fibroblast activation^62^. Thus, it is possible that FENDRR also reduces iron levels in macrophages and the decrease in iron levels in turn activates STAT1 signaling and thus promotes M1 macrophage polarization.

In summary, our results suggest that FENDRR promotes M1 macrophage polarization by modulating STAT1 activation pathway. Targeting FENDRR may provide a potential therapeutic benefit for the treatment of disorders associated with macrophage polarization.
